# Spontaneous Periodontitis Development in Diabetic Rats Involves an Unrestricted Expression of Inflammatory Cytokines and Tissue Destructive Factors in the Absence of Major Changes in Commensal Oral Microbiota

**DOI:** 10.1155/2012/356841

**Published:** 2012-05-07

**Authors:** Marcela Claudino, Gabriela Gennaro, Tania Mary Cestari, César Tadeu Spadella, Gustavo Pompermaier Garlet, Gerson Francisco Assis

**Affiliations:** ^1^Department of Biological Sciences, School of Dentistry of Bauru, Sao Paulo University (FOB/USP), Al. Dr Octávio Pinheiro Brisolla, 9-75, 17012-901 Bauru, SP, Brazil; ^2^Department of Surgery and Orthopedics, School of Medicine of Botucatu, Sao Paulo State University (UNESP), 18618-970 Botucatu, SP, Brazil

## Abstract

Diabetes mellitus is a heterogeneous group of disorders, in which hyperglycemia is a main feature. The objective was to evaluate the involvement of RAGE, inflammatory cytokines, and metalloproteinases in spontaneous periodontitis triggered by diabetes induction. Immunohistochemical procedures for MMP-2, MMP-9, TNF-**α**, IL-1**β**, IL-6, RANKL, and RAGE were performed in rats after 1, 3, 6, 9, and 12 months of diabetes induction. Total DNA was extracted from paraffin-embedded tissues and evaluated by Real-TimePCR for 16S total bacterial load and specific periodontopathogens. Our data did not demonstrate differences in microbiological patterns between groups. In diabetic groups, an increase in RAGE-positive cells was detected at 6, 9, and 12 months, while TNF-alpha-stained cells were more prevalent at 6 and 12 months. In experimental groups, IL-**β**-positive cells were increased after 12 months, IL-6 stained cells were increased at 9 and 12 months, and RANKL-positive cells at 9 months. Diabetes resulted in widespread expression of RAGE, followed by expression of proinflammatory mediators, without major alterations in oral microbial profile. The pervasive expression of cytokines suggests that spontaneous periodontitis development may be independent of microbial stimulation and may be triggered by diabetes-driven imbalance of homeostasis.

## 1. Introduction

Diabetes mellitus is a heterogeneous group of disorders that affect the metabolism of carbohydrates, lipids, and proteins, in which hyperglycemia is a main feature. The subsequent hyperglycemia has wide-ranging molecular and cellular effects, resulting in oxidative stress, upregulation of proinflammatory responses, and vascular changes that predispose individuals to the classic diabetes complications of cardiovascular disease, nephropathy, retinopathy, neuropathy, and atherosclerosis. Elevated blood glucose levels have been associated with diabetic complications throughout the acceleration of advanced glycation end products (AGEs) formation [1, 2]. The interaction between AGEs and their receptor RAGE generates reactive oxygen species and activates inflammatory signaling cascades, demonstrating the great influence of this interaction in the pathogenesis of several chronic diseases such as Alzheimer's disease, nondiabetic nephropathy, and chronic kidney disease [3–5]. In addition, AGE-RAGE interaction is also associated with diabetic complications [6, 7].

It also has been demonstrated that AGE-RAGE interaction results in increased levels of proinflammatory mediators such as tumoral necrosis factor alpha (TNF-alpha), interleukin-1beta (IL-1beta), interleukin-6 (IL-6), and matrix metalloproteinases (MMPs) [8, 9]. In fact, previous studies [5, 10, 11] revealed that higher levels of inflammatory mediators are related to diabetic complications such as nephropathy, neuropathy, retinopathy, and cardiovascular diseases. Then, the central role of AGEs and RAGE in maintaining perpetuated cell activation is demonstrated in various therapeutic attempts to block RAGE or their ligands [12, 13].

Among the pathologies triggered or exacerbated by diabetes, the effect of chronic hyperglycemic in periodontal environment has been characterized by an increase in periodontitis prevalence and severity. In this way, periodontal disease has been termed the sixth complication of diabetes [14–16]. Several mechanisms have been proposed to explain the interactions between diabetes and periodontal diseases. These potential mechanisms are strikingly similar to those associated with the established complications of chronic diabetes, suggesting that increased severity of periodontal disease in presence of diabetes was due to an exacerbated inflammatory response triggered by AGEs [17, 18]. In fact, clinical and experimental studies [15, 19] have been demonstrating an association between diabetes and increased expression of inflammatory mediators, osteoclastogenic factors, and matrix metalloproteases. Some studies [20, 21] also suggest that diabetes interfere in host defense mechanisms such as chemotaxis, adherence, phagocytosis, and apoptosis, contributing to tissue destruction. 

These studies demonstrated that diabetes increases the severity of periodontal disease previously induced by bacterial inoculation or by silk ligatures [22, 23]. However, a previous study [24] demonstrated that a chronic hyperglycemic environment in periodontal tissue, even in absence of external stimuli such as bacterial inoculation or silk ligatures, results in the clear establishment and progression of periodontal disease. In fact, previous reports suggested that diabetes not only exacerbate the periodontitis severity but also can trigger periodontal disease induction [25]. Interestingly, it has been suggested that the inflammatory/immune deregulation caused by diabetes could trigger periodontitis development in response to the commensal subgingival microflora [9, 16, 23]. However, the mechanisms involved in the spontaneous onset of periodontitis after diabetes induction remains unknown.

In this context, the purpose of this study was to investigate the potential mechanisms involved in the spontaneous onset of diabetes-triggered periodontitis through the quantitative and spatial evaluation of RAGE, TNF-alpha, IL-1beta, IL-6, RANKL, MMP-2, MMP-9 immunostaining patterns, and also the monitoring of oral microbiota load, in periodontal tissue in rats after 1, 3, 6, 9, and 12 months of diabetes induction.

## 2. Material and Methods

### 2.1. Induction of Diabetes and Collection of Samples

Adult male Wistar rats, 10–12 weeks old, weighing approximately 250 g, were submitted to diabetes induction by intravenous administration of alloxan (Sigma Chemical Co., St Louis, EUA) in a single dose of 42 mg/kg of body weight into the caudal vein. Only rats showing two successive determinations of blood glucose levels (7 and 14 days after alloxan injection) greater than 250 mg/dL were considered diabetic and included in the experiment. The glucose levels during the experiment were typically 400–450 mg/dL in diabetic rats, while serum glucose levels of control rats ranged from 90 to 130 mg/dL. The animals were housed in metabolic cages in groups of four per cage and fed a standard rat chow and water *ad libitum*. Study protocol was approved by the Experimental Committee of Bauru School of Dentistry, São Paulo State University (033/2007). Each diabetic and control group was composed of 25 animals, subdivided into 5 subgroups with 5 rats each, analyzed at 1, 3, 6, 9, and 12 months after diabetes induction. On their respective dates, the animals were sacrificed with overdose of sodium pentobarbital (Cristália Chemical Products-Itapira/SP) and the hemimandibles were removed and fixed in 10% formalin solution for 7 days.

### 2.2. Histological and Immunohistochemical Analyses

The hemimandibles were demineralized for 8 weeks in 4.13% EDTA, washed, dehydrated, and embedded in paraffin. Slides of 5 *μ*m thick serial sections of the each hemimandible were obtained (3 semi-serial sections for each protein/antibody studied) and submitted to immunohistochemical procedures. Immunohistochemistry was performed using the Streptavidin-Biotin/HRP Complex method.

Paraffin sections (3 semi-serial section of each animal) were deparaffinized with xylol, dehydrated in decreasing alcohol solutions, and the endogenous peroxidase was blocked with 3% hydrogen peroxide solution in phosphate-buffered saline (PBS) for 30 minutes at room temperature. For antigen retrieval, sections were treated with 0.5% pepsin (IL-1beta, TNF-alpha, IL-6 and RAGE antibodies) and 0.4 mg/mL of proteinase K (RANKL antibody) for 20 minutes at room temperature. In MMP-2 and MMP-9 reactions, sections were submitted to sodium citrate (10 mM; pH 6.0) for 20 minutes at 95°C. After antigen retrieval, sections were incubated in 7% free-fat milk for 40 minutes at room temperature.

A specific primary antibodies for MMP-9 (sc-6840-Santa Cruz Biotechnology, Inc., Santa Cruz, CA, USA) at 1 : 200 dilution; MMP-2 (sc-58386-Santa Cruz Biotechnology, Inc.), at 1 : 100 dilution; RANKL (sc-7628-Santa Cruz Biotechnology, Inc.), at 1 : 50 dilution; RAGE (ab3611-Abcam Inc., Cambridge, USA), at 1 : 50 dilution; TNF-alpha (sc-1348-Santa Cruz Biotechnology, Inc.), at 1 : 50 dilution; IL-1beta (sc-7884-Santa Cruz Biotechnology, Inc.), at 1 : 50 dilution; IL-6 (IL-6-Santa Cruz Biotechnology, Inc.), at 1 : 50 dilution were applied and incubated for 2 hours at room temperature in a humidified chamber.

After washing, the sections were incubated with biotin-conjugated secondary antibody (sc-2774 and sc-2040-Santa Cruz Biotechnology, Inc.) for 1 h at room temperature followed by streptavidin/peroxidase complex (Dakocytomation, Glostrup, Denmark) for 20 minutes at room temperature. Immunoreactivity was developed using 3,30-diaminobenzidine, for 5 min (DakoCytomation, Glostrup, Denmark), counterstained with Harris's hematoxylin and mounted. All rinses were performed three times in 0.1% PBS/Triton-X. Negative controls were performed replacing the primaries antibodies for the same PBS/BSA solution. Periodontal disease tissue was used as a positive control for TNF-alpha, IL-1beta, IL-6, and RANKL [26]. Accordingly to fabricant instruction, rat lung tissue exposed to tobacco was used as positive control for MMP-2, MMP-9, and RAGE.

All histological sections were identified with a random numerical sequence in order to codify experimental periods and groups during the analysis procedures. A single calibrated investigator analyzed the sections with a binocular microscope (Olympus Optical Co., Tokyo, Japan). Morphometrical measurements were obtained using a 100x immersion objective and a Zeiss kpl 8x eyepiece containing a Zeiss II integration grid (Carl Zeiss Jena GmbH, Jena, Germany) with 10 parallel lines and 100 points in a quadrangular area. The grid image was successively superimposed on approximately 15 histological fields per histological section, comprising all the periodontal area, from the junctional epithelium to the root apex of mesial face of lower first molar mesial root. In each field, immunostained cells are counted and results were presented as number of immunolabeled cells per mm^2^ of tissue in each examined group.

### 2.3. DNA Extraction and Real-Time PCR Reactions

In order to allow the quantification of the bacteria, which potentially invaded the host tissues, we performed the extraction of bacterial DNA. Histological sections formalin-fixed (3 semi-serial section of each animal), paraffin-embedded tissue sections comprising the teeth and surrounding structures were submitted to DNA extraction with QIAamp DNA FFPE Tissue Kit (QUIAGEN) following manufacturer instructions. Bacterial load (16S) was quantified by Real-Time-PCR performed in a MiniOpticon system (BioRad, Hercules, CA, USA), using SybrGreenMasterMix (Invitrogen), with 100 nM specific primers and 5 ng of DNA in each reaction, as previously described [27]. 

### 2.4. Statistical Analyses

A two-way ANOVA was employed to analyze data from all groups, followed by one-way ANOVA and Tukey's test to determine significant differences within the same group related to time. The significance level was always set at *P* < 0.05, and all calculations were performed using GraphPad program Prism 3.0 (GraphPad Software Inc, EUA).

## 3. Results

**Figure 1:**
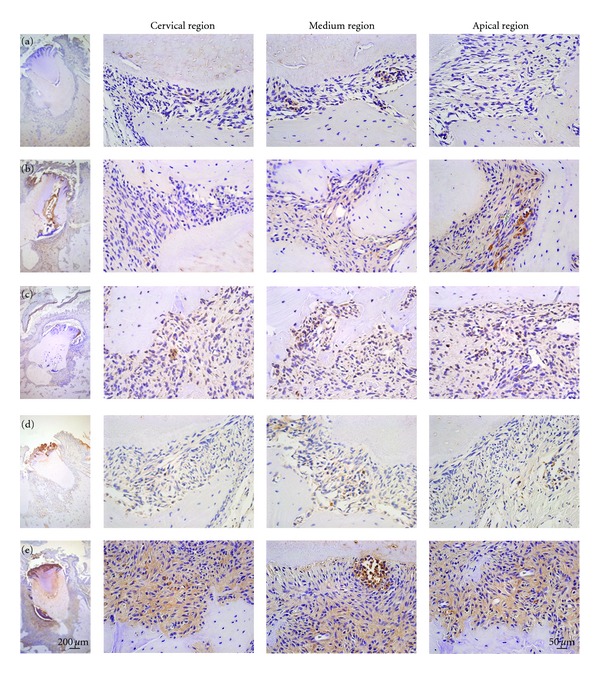
Increased number of immunoreactive cells after 9 months of diabetes induction in rats. Histological sections of periodontal tissues were submitted to immunohistochemical analyses for RAGE (a), TNF-alpha (b), IL-1beta (c), IL-6 (d), and RANKL (e) after 9 months of diabetes induction in rats. Observe the presence of immunoreactive cells in all regions of periodontium (cervical, medium, and apical regions), being not limited to the gingival area.

Our immunohistochemical results did not show significant differences between control and experimental groups after 1 and 3 months of diabetes induction. However, differences were detected after 6, 9, and 12 months regarding to RAGE, TNF-alpha, IL-1beta, IL-6, and RANKL, as illustrated in Figure 1. The immunoreactive cells were detected in different regions of periodontal tissues with different morphologies. Considering that we did not observe a focal distribution of immunostained cells, we performed a quantification comprising all regions of periodontium in order to quantify the number of immunoreactive cells.

**Figure 2:**
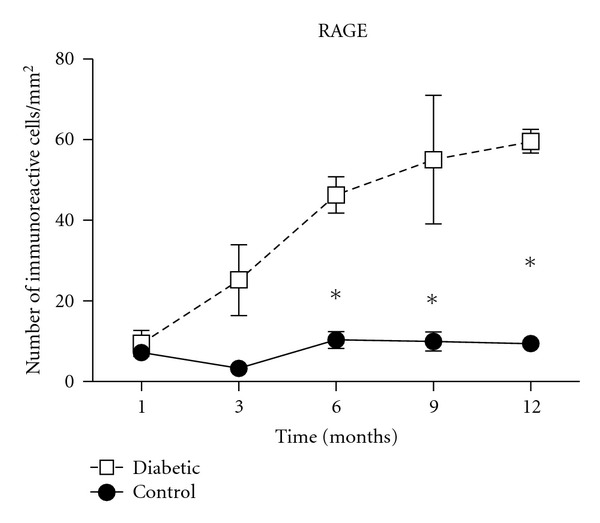
Increased number of RAGE-positive cells in diabetic groups. Histological sections of periodontal tissues were submitted to immunohistochemical analyses for RAGE after 1, 3, 6, 9, and 12 months of diabetes induction. The results are presented as mean ± SD of immunoreactive cells per mm^2^ in 3 semiserial section of each animal, *P < 0.05, one-way analysis of variance (ANOVA) followed by Tukey's test.

Regarding to RAGE, we initially detected increased number of RAGE-positive cells in diabetic groups after 6 (*P* < 0.001), 9 (*P* < 0.01), and 12 (*P* < 0.01) months when compared to control group (Figure 2). Evaluating different periods of control group, we did not found any differences in immunoreactivity patterns. Nevertheless, diabetic groups revealed changes in the number of RAGE-positive cells among periods of experimental design.

**Figure 3:**
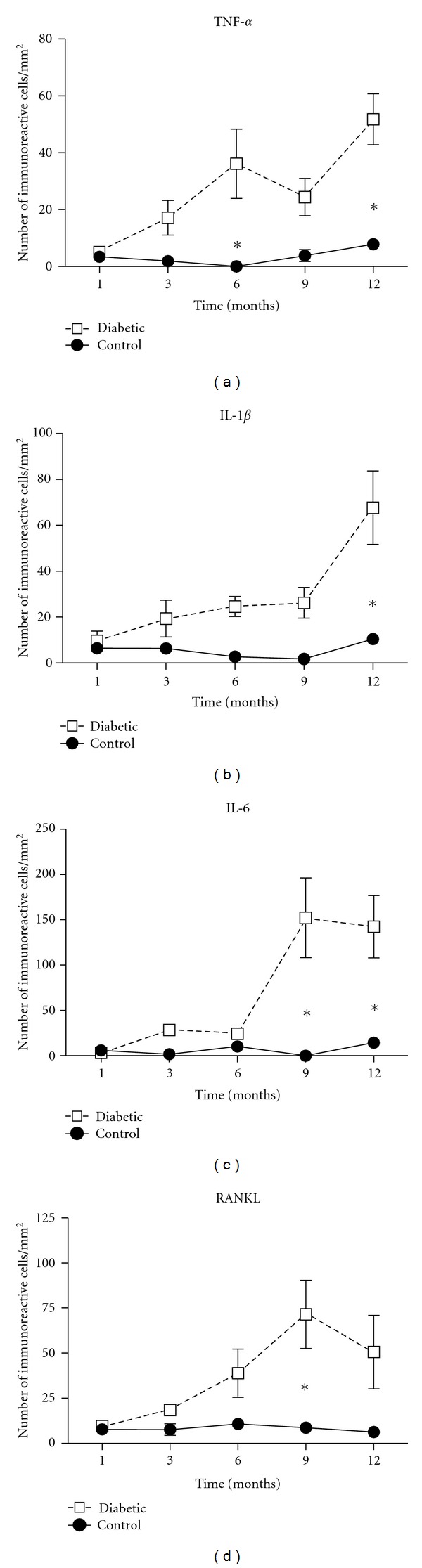
Increased number of immunoreactive cells for TNF-alpha, IL-1beta, IL-6, and RANKL in diabetic groups. After 1, 3, 6, 9, and 12 months of diabetes induction, histological sections of periodontal tissues were submitted to immunohistochemical analyses for TNF-alpha, IL-1beta, IL-6, and RANKL. The results are presented as mean ± SD of immunoreactive cells per mm^2^ in 3 semiserial section of each animal, *P < 0.05, one-way analysis of variance (ANOVA) followed by Tukey's test.

Our next step was to evaluate the number of positive cells regarding to proinflammatory cytokines such as TNF-alpha, IL-1beta, and IL-6 (Figure 3). Evaluating the number of TNF-alpha-positive cells, we found a statistical significant increase in experimental groups after 6 (*P* < 0.05) and 12 months (*P* < 0.001) when compared to their respective control groups (Figure 3). Different time points of control groups did not show any differences, while diabetic groups revealed a trend of increase (*P* > 0.05) after 3 months. This difference became more pronounced at later periods, being statistically significant after 6 and 12 months.

Moreover, the number of IL-1beta-positive cells was increased only after 12 months (*P* < 0.001) in diabetic group (Figure 3). In control groups, few immunoreactive cells were detected in all periods; however, experimental groups revealed a trend of increase (*P* > 0.05) at 3, 6, and 9 months. In IL-6 experiments, we observed an increase in the number of immunoreactive cells in diabetic groups at 9 (*P* < 0.001) and 12 (*P* < 0.01) months compared to control groups (Figure 3).

Regarding to RANKL, diabetic groups presented the same pattern as control groups in initial periods (1 and 3 months). However, a trend of increase (*P* > 0.05) was observed in next periods, being statistically significant after 9 months of diabetes induction (*P* < 0.001) (Figure 3).

**Figure 4:**
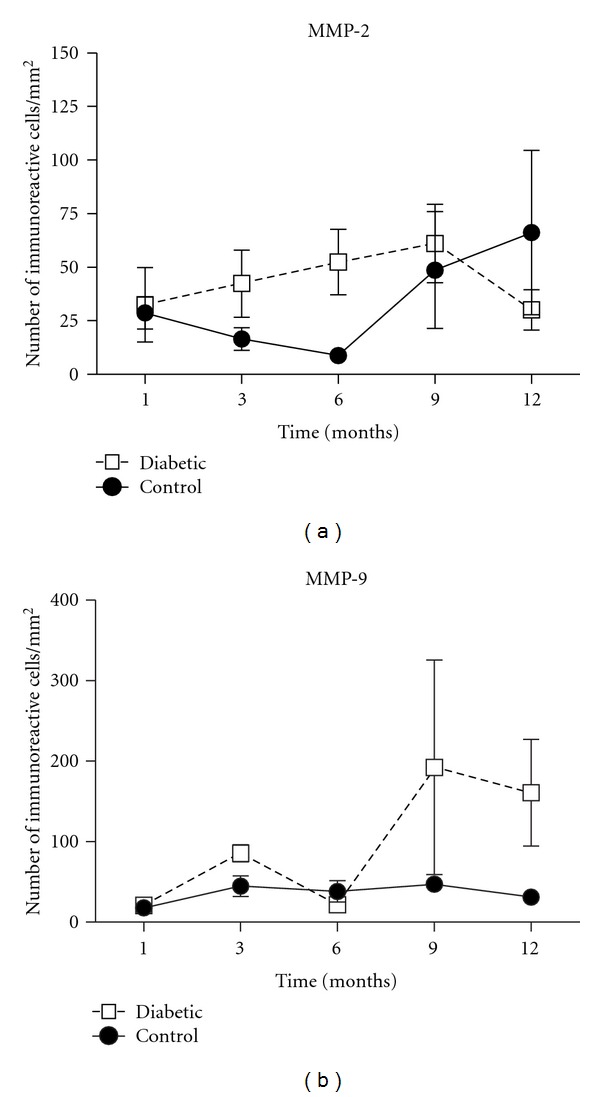
Absence of differences in the number of MMP-2 and MMP-9-positive cells between control and diabetic groups. After 1, 3, 6, 9, and 12 months of diabetes induction, histological sections of periodontal tissues were submitted to immunohistochemical analyses for MMP-2 (a) and MMP-9 (b). The results are presented as mean ± SD of immunoreactive cells per mm^2^ in 3 semiserial section of each animal, *P < 0.05, one-way analysis of variance (ANOVA) followed by Tukey's test.

In addition, we did not observe significant statistical differences between control and experimental groups in MMP-2 and MMP-9 at any timepoints (Figure 4). We observed few MMP-2-positive cells in initial periods with a trend of increase (*P* > 0.05) in control groups after 9 and 12 months. In experimental group, it was detected a slight increase in the number of MMP-2-positive cells, followed by a trend to reduction in the period of 12 months. We also detected a trend of increase (*P* > 0.05) in the number of MMP-9-positive cells in diabetic group after 12 months of diabetes induction.

These results are the mean value of 15 histological fields per histological section, comprising all the periodontal area from the gingival crest to the root apex. When the results were individually analyzed by a subdivision of periodontal area in cervical, medium, and apical regions (5 histological fields in each region), no significant differences were found in the distribution of immunoreactive cells (data not shown).

**Figure 5:**
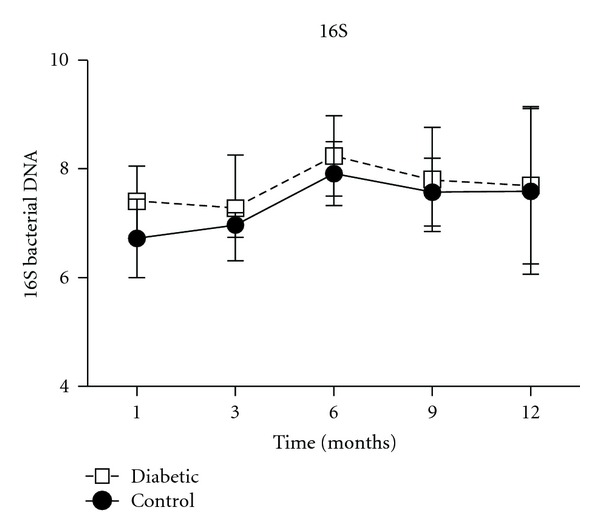
Variations in the number of immunoreactive cells is not associated with bacterial load. Bacterial load (16S) in periodontal tissues was quantified by Real-TimePCR, using SyberGreen system and normalized by tissue weight. One-way analysis of variance (ANOVA) followed by Tukey's test.

We also evaluated the overall microbial load in oral cavity, and the results demonstrated that the levels of 16S bacterial DNA were similar in all the periods evaluated in both diabetic and control groups (Figure 5). The DNA samples extracted from periodontal tissue sections were also investigated regarding the presence of classic periodontopathogens (*Actinomyces actinomycetemcomitans*, *Porphyromonas gingivalis*, *Prevotella nigrescens, Tannerella forsythia,* and *Treponema denticola*) but no positive samples were observed in any period of both diabetic and control groups (data not shown).

## 4. Discussion

It is well established that periodontal disease is more severe and frequent in presence of diabetes [14–16], being associated with higher levels of proinflammatory cytokines, osteoclastogenic factors, and metalloproteases [19, 26, 28]. While the modulation of the established periodontal disease by diabetes seems to be a consensus in the scientific literature, the potential coinduction of periodontal disease by diabetes recently demonstrated [14, 25] remains almost unknown. In this context, the co-induction hypothesis proposed is characterized by a “two-hit model,” where the “first hit” is supposed to be provided by the oral microflora. In this way, the “second hit” is represented by the exacerbated inflammatory response driven by systemic diseases such diabetes [25].

In accordance with this coinduction hypothesis [25], our previous results demonstrated that diabetes induction trigger alterations, which are typical of periodontal diseases even in the absence of periodontitis induction [24]. However, in our initial report, no attempts to characterize possible changes in the overall oral microbial profile as well to evaluate potential mechanisms underlying spontaneous periodontitis development were conducted. Then, the aim of this study was to investigate the possible microbiological changes and the spatial distribution and the number of immunostained cells to RAGE, TNF-alpha, IL-1beta, IL-6, RANKL and MMP-2, and MMP-9, in periodontal tissue after diabetes induction.

 Initially, we investigated the overall bacterial load in oral cavity, represented by the 16S bacterial DNA levels, and our results also did not show variations in periodontal bacterial load, suggesting the absence of quantitative differences in the biofilm in both groups. Also, we investigated the presence of classical periodontopathogens such as *Actinomyces actinomycetemcomitans*, *Porphyromonas gingivalis*, *Prevotella nigrescens, Tannerella forsythia,* and *Treponema denticola*, but no positive samples were found along the disease development in diabetic group nor in control animals. In this way, our data suggest that the presence of diabetes did not affect qualitative and quantitative pattern of dental biofilm.

Interestingly, previous studies showed that exacerbated periodontal disease associated to diabetes is caused by classical periodontopathogens, but it is important to consider that the aggravation of existing disease is distinct of the coinduction situation, where classical periodontopathogens were supposed to be absent in health conditions [29]. Then, the establishment of periodontal disease seems to be a result of an exacerbated response against a conventional stimulus provided by the periodontopathogenic subgingival microflora [25].

Thus, our next step was to investigate the possible effects of diabetes over host response throughout immunohistochemical evaluations. Interestingly, we observed an increase in the number of immunostained cells to RAGE after 6, 9, and 12 months of diabetes induction, confirming the involvement of these receptors. In agreement, it has been found an increase in RAGE expression in the gingival tissues of diabetic patients compared to nondiabetic patients with periodontal disease [30, 31]. Likewise, diabetic mice showed an increased AGEs deposition and RAGE expression in the gingival tissues [32]. In addition, blockade of AGE-RAGE interaction with soluble RAGE receptor in diabetic mice decreased alveolar bone resorption and expression of cytokines and metalloproteinases [13, 18]. Likewise, RAGE was demonstrated to mediate osteoclasts formation and activation with consequent bone resorption and decrease of bone density, and to upregulate the expression of proinflammatory cytokines [33].

Accordingly, we observed an increased number of TNF-alpha, IL-1beta, and IL-6-positive cells in diabetic groups. Regarding to TNF-alpha, we found an increase in the number of TNF-alpha-positive cells after 6 and 12 months of diabetes induction. In accordance with our data, higher serum levels of this cytokine have been detected in patients with poorly controlled diabetes when compared to healthy patients [34, 35]. Also, increased expression of TNF-alpha was also observed in diabetic mice after oral inoculation of *Porphyromonas gingivalis *[23]. In fact, macrophages from diabetic patients had greater ability to produce TNF-alpha when compared to macrophages from nondiabetic patients [19]. It also has been demonstrated that LPS stimulation resulted in a trend of increase in TNF-alpha, IL-1beta, and IL-6 expression *in vitro *[36].

Regarding IL-1beta, we observed an increased number of immunostaining cells after 12 months of diabetes induction. Other studies also reported increased expression of IL-1beta in diabetic patients with periodontal disease [19, 37]. Conversely, it has been described that diabetic patients with periodontal disease presented decreased levels of IL-1 when compared to non-diabetic patients [28], while other authors did not found differences regarding to cytokines expression in diabetic and non-diabetic patients with periodontitis [38]. It is possible that increased TNF-alpha and IL-1beta levels affect the expression of IL-6, considering that we detected an increase in the number of immunostained cells to IL-6 after 9 and 12 months of diabetes induction. Considering that an augment was observed in IL-6 expression in cultures of gingival fibroblasts stimulated with TNF-alpha and IL-1beta, it has been suggested that exacerbated expression of TNF-alpha and IL-1beta regulate IL-6 expression [39]. According to our results, increased expression of IL-6 has been described in diabetic patients with periodontitis [35]. Similarly, other authors demonstrated a trend to increased IL-6 levels in diabetic subjects with periodontal disease [37, 38].

 It is well established that the proinflammatory cytokines above discussed present a great influence in osteoclastogenesis and characteristically affect RANKL expression [39]. Indeed, our data showed increased number of RANKL-positive cells after 9 months of diabetes induction. Although contradictory results have been demonstrated that diabetes did not affect RANKL expression [38], other authors showed that diabetic mice presented decreased bone density accompanied by higher levels of RANKL, IL-1, and IL-6 [40]. Then, it has been described that increased expression of RANKL axis suggesting that RANK/RANKL/OPG is involved in the molecular basis of the association between diabetes and periodontal disease [41].

In addition, other authors have evaluated the influence of diabetes in MMPs expression, demonstrating higher levels of MMP-8 and MMP-9 in periodontal tissue of diabetic patients with periodontitis [42]. However, other authors did not found differences in MMP-1 and MMP-8 expression in periodontal tissue and gingival crevicular fluid of diabetic and nondiabetic patients, both with periodontal disease [37]. Although our data did not show significant differences in MMP-2 and MMP-9 immunoreactivities, we detected a trend of increase in the number of MMP-9-positive cells in rats after 9 and 12 months of diabetes induction. Accordingly, differences in MMP-2 levels in diabetic rats with ligature-induced periodontal disease was not observed; however, higher levels of MMP-9 were detected [22]. Other authors demonstrated that, even in the absence of periodontal disease, MMP-8 and MMP-9 expression was increased in periodontal tissue of diabetic rats, while no differences were observed regarding to MMP-2 and MMP-3 between control and diabetic groups [43]. In addition, it has been demonstrated that the amount of glucose affects the MMP-1 expression *in vitro* [44].

 Based on these data, it is possible to suggest that diabetes affect expression of mediators related to establishment and progression of periodontal disease. Although the mechanisms remain poorly understood, the coinduction of periodontitis by diabetes is supposed to be the result of the RAGE/AGEs interaction, which results in the generation of a proinflammatory environment [12, 13, 18, 31]. Accordingly, the upregulation of RAGE immunoreactive cells number precedes the increase of proinflammatory cytokines and RANKL expression. Interestingly, these changes seem to not be accompanied by a marked change in the overall bacterial load in oral cavity. While this data do not support the statement that the coinduction is independent of the microbial stimuli, the pattern of immunostaining for all the factors investigated demonstrate that the expression of RAGE and cytokines is not limited to the gingival area, where the periodontal biofilm-stimulated responses characteristically are localized. Therefore, it is possible to suggest that factors other than the oral microflora could act as triggers, or the “second hit”, in distinct areas of the periodontal tissues.

In this context, it is interesting to consider that RAGE signalling results in an upregulation of TLR2 and TLR4 receptors, resulting in an MyD88-dependent signaling and subsequent activation of NF-kappaB, increasing the expression of inflammatory cytokines such as IL-6, IL-1, and TNF-alpha [45]. Classically, the pathogen-associated molecular patterns (PAMPs) of commensal subgingival flora are recognized as the typical ligands of TLRs in oral environment [46]. However, the presence of high concentration of AGEs, as a consequence of chronic hyperglycemia, may act as an endogenous danger signal, related to damage-associated molecular pattern (DAMPs), which can also trigger the TLR/MyD88/NF-kappaB pathway. The identification of this “danger signal” by atypical pattern-recognition receptors such as RAGE seems to stimulate and exacerbate the conventional response.

In this way, the initial inflammatory and immune response could be sustained throughout the interaction between the high concentrations of AGEs and atypical pattern-recognition receptors such as RAGE. In this way, DAMPs could not only activate the response but could also influence the pattern of immune and inflammatory response, exacerbating these responses even in the absence of classic PAMPs in areas of periodontal tissue distant from the gingival biofilm. The absence of differences could be explained based on RAGE expression, which seems to be upregulated in presence of chronic hyperglycemic environment.

Considering that higher levels of glycemia affect all periodontal regions, not only the regions in contact with periodontopathogens, the absence of differences in the distribution of immunoreactive cells could be explained based on the central role of AGE-RAGE interaction, whose could be detected in all regions of periodontal tissues. In fact, this mechanism seems to be associated with arthritis development, a chronic systemic disease related to altered patterns of bone resorption, as observed in periodontitis [47–49].

 Taken together, diabetes seems to be associated with host response dysfunction, which exacerbates the expression and/or activation of intracellular signaling molecules, resulting in upregulation of inflammatory mediators. Our results demonstrated that diabetes may trigger periodontal disease induction throughout the dysregulation of immune and inflammatory response against commensal periodopathogenic microbiota. In fact, these biological events may partially explain the increased severity of periodontal disease and decreased ability to repair tissue in presence of diabetes. However, further studies must be carried out to improve knowledge on the interaction between diabetes and periodontal diseases, which may serve as a basis for the development of more effective strategies for prevention and treatment of periodontitis in diabetic patients.
